# First case of an invasive *Bacteroides dorei* infection detected in a patient with a mycotic aortic aneurysm—raising a rebellion of major indigenous bacteria in humans: a case report and review

**DOI:** 10.1186/s12879-021-06345-8

**Published:** 2021-06-30

**Authors:** Takayuki Matsuoka, Takuya Shimizu, Tadanori Minagawa, Wakiko Hiranuma, Miki Takeda, Risako Kakuta, Shunsuke Kawamoto

**Affiliations:** 1 Department of Cardiovascular Surgery, Tohoku Medical and Pharmaceutical University, 1-12-1 Fukumuro, Miyagino Ward, Sendai, Miyagi 983-8512 Japan; 2 Department of Otolaryngology-Head and Neck Surgery, Tohoku University Graduate School of Medicine, Sendai, Japan

**Keywords:** Case report, *Bacteroides dorei*, Mycotic aortic aneurysm, 16S rRNA gene sequencing, Microbiota, Indigenous intestinal bacteria, Mass spectrometry

## Abstract

**Background:**

*Bacteroides dorei* is an anaerobic gram-negative bacterium first described in 2006. Because of the high similarity in mass spectra between *B. dorei* and *Bacteroides vulgatus*, discriminating between these species is arduous in clinical practice. In recent decades, 16S rRNA gene sequencing has been a complementary method for distinguishing taxonomically close bacteria, including *B. dorei* and *B. vulgatus*, at the genus and species levels*.* Consequently, *B. dorei* has been shown to contribute to some diseases, including type 1 autoimmune diabetes mellitus and atherosclerotic diseases. However, there are no reports on invasive infectious diseases caused by *B. dorei*. This report describes the first case of direct invasion and colonisation of human tissue by *B. dorei*, thus providing a warning regarding the previously proposed application of *B. dorei* as a live biotherapeutic for atherosclerotic diseases.

**Case presentation:**

A 78-year-old Japanese man complained of intermittent chest/back pain and was diagnosed with a mycotic thoracic aortic aneurysm by enhanced computed tomography on admission. Despite strict blood pressure control and empirical antibiotic therapy, the patient’s condition worsened. To prevent aneurysmal rupture and eliminate infectious foci, the patient underwent surgical treatment. The resected specimen was subjected to tissue culture and 16S rRNA gene sequencing analysis to identify pathogenic bacteria. A few days after the surgery, culture and sequencing results revealed that the pathogen was *B. dorei*/*B. vulgatus* and *B. dorei*, respectively. The patient was successfully treated with appropriate antibacterial therapy and after improvement, was transferred to another hospital for rehabilitation on postoperative day 34. There was no recurrence of infection or aneurysm after the patient transfer.

**Conclusions:**

This report describes the first case of invasive infectious disease caused by *B. dorei*, casting a shadow over its utilisation as a probiotic for atherosclerotic diseases.

**Supplementary Information:**

The online version contains supplementary material available at 10.1186/s12879-021-06345-8.

## Background

*Bacteroides dorei* is a gram-negative anaerobic rod that is generally isolated from the human and animal gastrointestinal tract [1] and is one of the cardinal indigenous bacteria in humans [2]. In clinical settings, matrix-assisted laser desorption ionisation time-of-flight mass spectrometry (MALDI-TOF MS) analysis has prominently contributed to the identification of pathogenic bacteria and fungi, and its identification accuracy was estimated to be as high as 84% for species and 92% for genera [3]. However, it is known that this methodology has some limitations for taxonomically close species or anaerobic bacteria. Some researchers have shown a low performance of MALDI-TOF MS in the identification of anaerobic bacteria, partially due to insufficient commercial mass spectral reference libraries, resulting in the misidentification of pathogens, e.g. *B. dorei* as *Bacteroides vulgatus* [4, 5]. In the last decade, 16S rRNA gene sequencing has been applied for bacterial identification in many facilities. This polymerase chain reaction (PCR)-based method is highly efficient for discriminating phylogenetically close bacteria at the genus and species levels. Therefore, it is relevant as a complementary method for blood culture, MALDI-TOF MS, and conventional phenotypic screening tests for the identification of bacterial pathogens [6].

Recently, a role for *B. dorei* as an immunomodulator in autoimmune diseases has been uncovered [7]. Other reports have also shown that the alteration of the abundance of *Bacteroides* species in the intestinal microbiota was associated with the susceptibility to autoimmune or atherosclerotic diseases. It is thus assumed that these bacteria may be used as therapeutic targets for these diseases, especially in the form of probiotics or biotherapy [8]. However, there are almost no reports indicating direct involvement of *B. dorei* in the pathogenesis of infections.

This report describes the first case of *B. dorei* as a pathogen of an invasive infectious disease, suggesting the need for caution in the use of *B. dorei* as a biotherapeutic.

## Case presentation

**Fig. 1:**
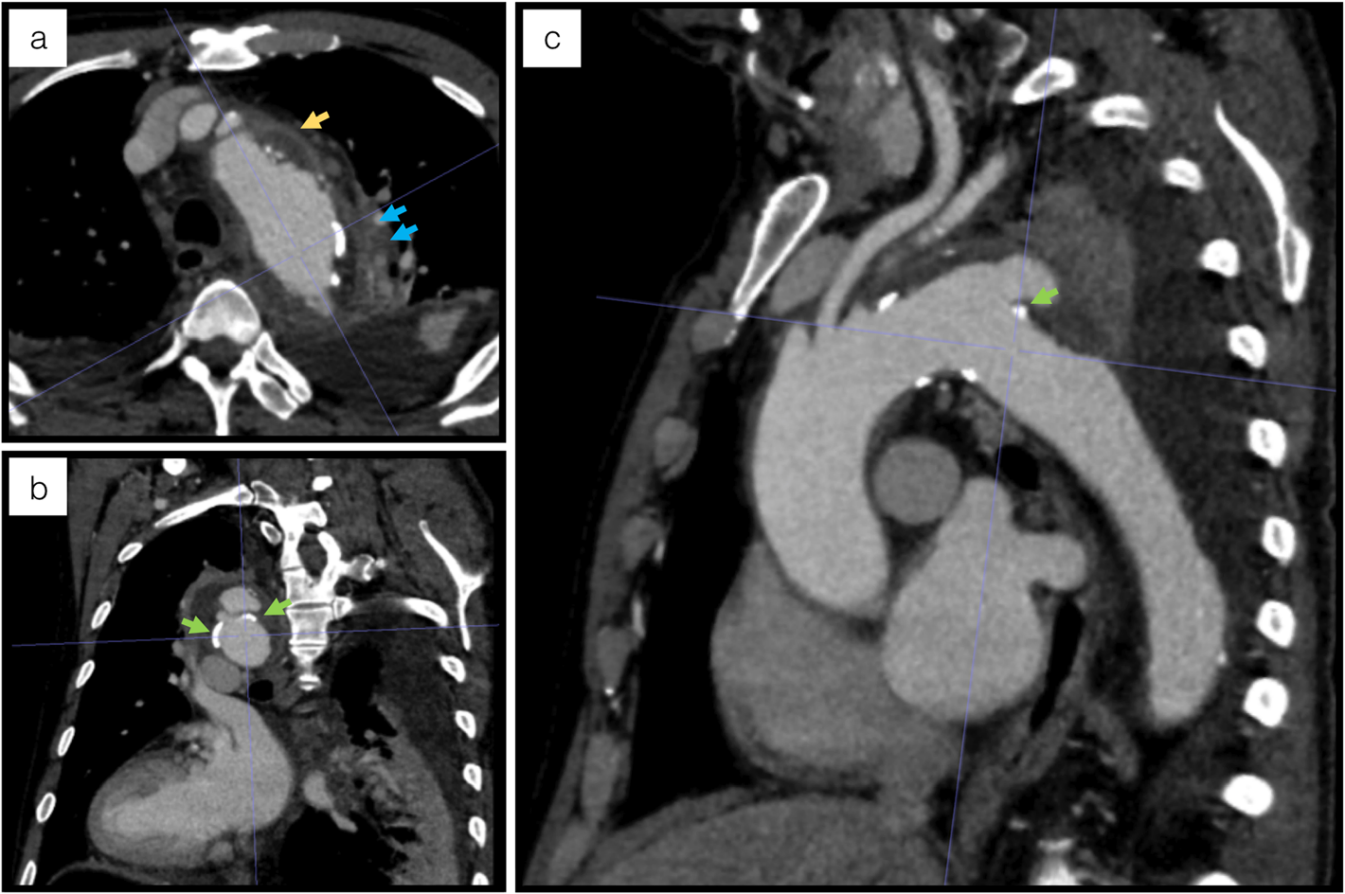
Contrast-enhanced computed tomography scan of the patient on admission. **a** Axial view shows a distal aortic arch aneurysm with a perianeurysmal fluid density collection (yellow arrow) and periaortic inflammatory changes (blue arrows). Coronal (**b**) and sagittal (**c**) views show the dislocation of intimal calcification (green arrows) and the appearance of aortic dissection

**Fig. 2:**
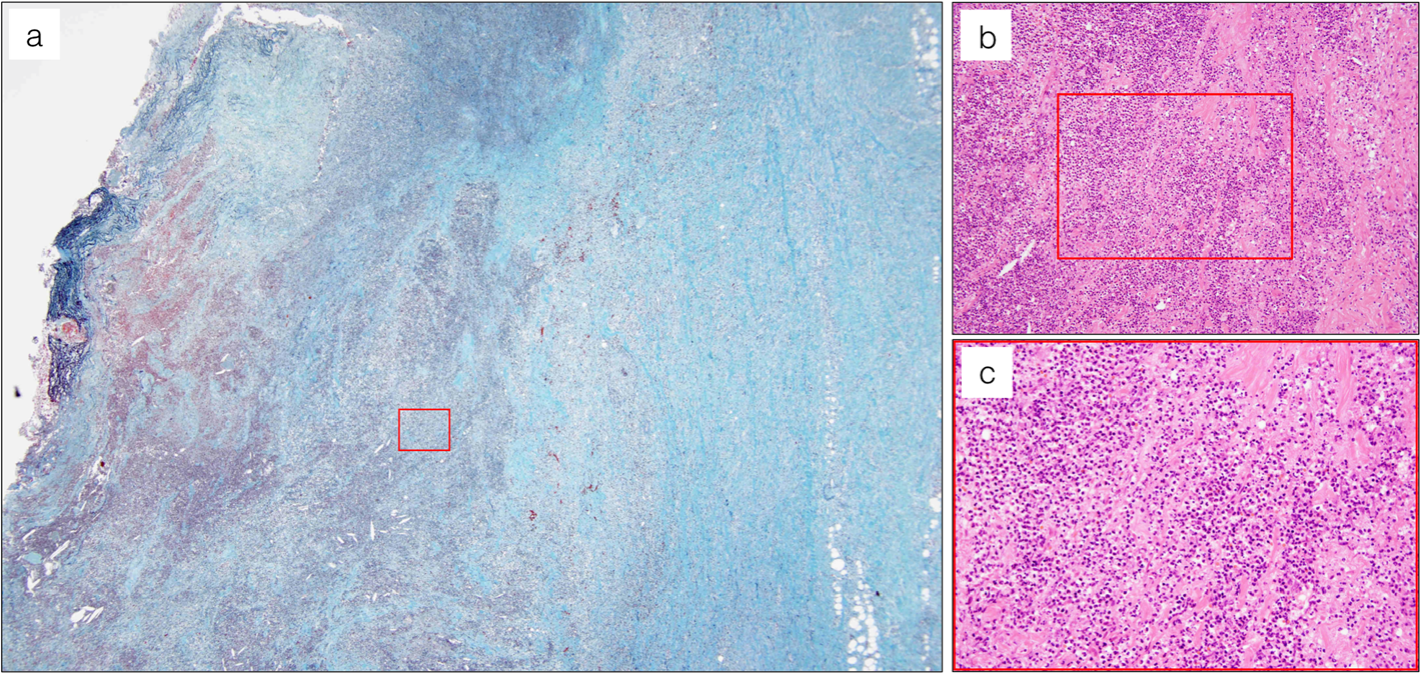
Pathological examination of the resected aorta. Microscopic examination of the resected specimen was conducted with Elastica-Masson staining (**a**) and haematoxylin and eosin staining (**b**, **c**). **a** Significant immunocyte infiltration is observed in the sub-adventitial layer, depicting purulent inflammation. **b**, **c** Abundant neutrophils infiltrated into the intramural area of the infected arterial wall, forming an abscess

**Fig. 3:**
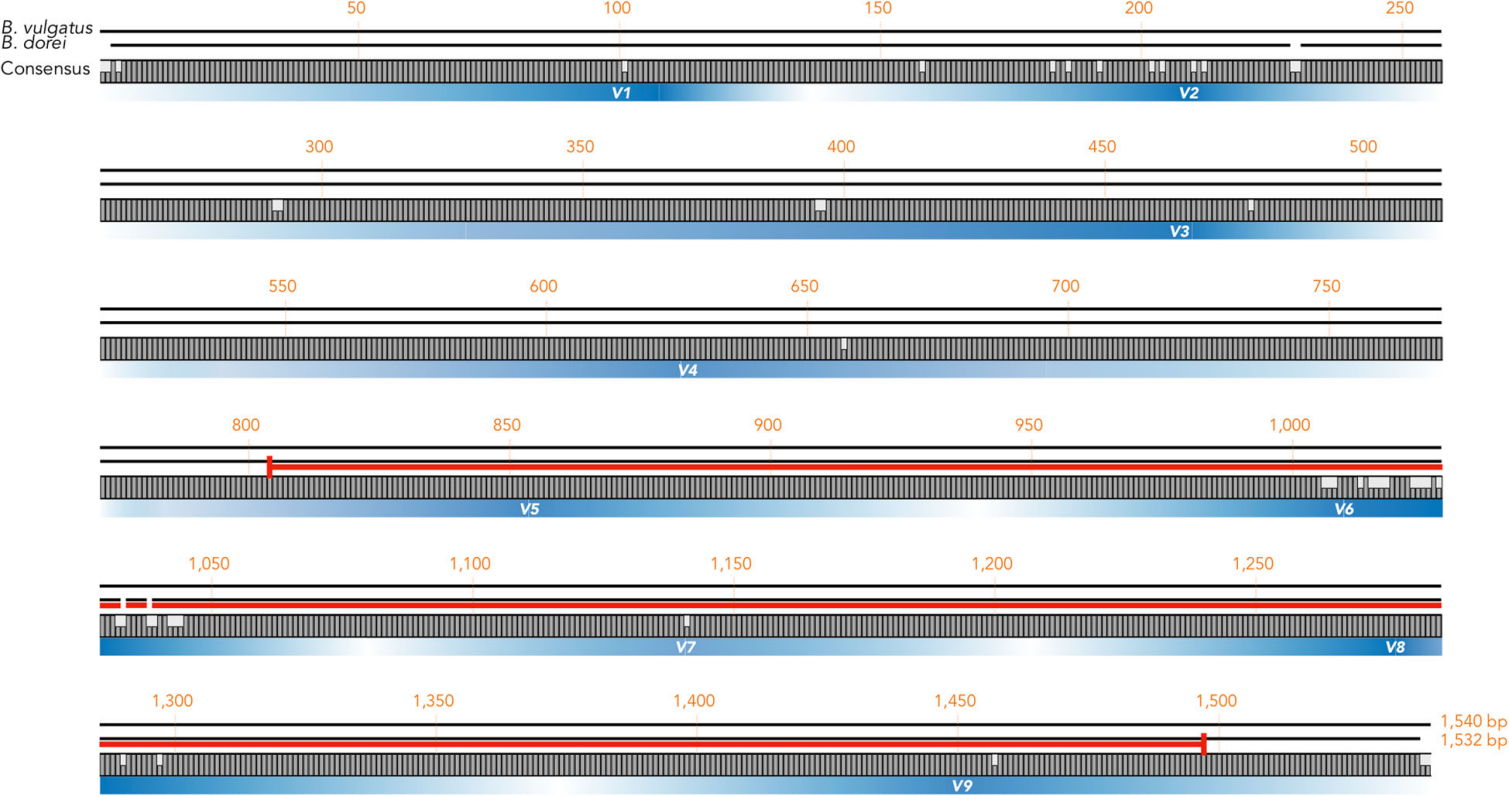
16S rRNA gene sequence analysis for the identification of pathogenic bacteria. The *Bacteroides dorei* (DSM 17855) and *Bacteroides vulgatus* (ATCC 8482^T^) 16S rRNA gene sequences were aligned based on the consensus, with a concordance rate as high as 97%. V1-V9 represent the variable regions in the gene sequence, and the red bar indicates the sequencing range for identifying pathogens in this study. *B. dorei* was identified as the pathogen in this patient, with 100% sequence identity with the available *B. dorei* sequence

A 78-year-old Japanese man presented with intermittent chest/back pain and was admitted to our hospital with a suspicion of a mycotic thoracic aortic aneurysm. The patient had a medical history of hypertension, type 2 diabetes mellitus (DM) (glycated haemoglobin level of 7.9% on admission), and dyslipidaemia for more than 10 years but no relevant family history and history of smoking, drug abuse or alcohol consumption. On admission, the patient was afebrile and showed unremarkable manifestations or physical findings, except for back dysphoria. A contrast-enhanced computed tomography (eCT) scan confirmed the appearance of a mycotic thoracic aortic aneurysm and dissection at the distal arch, with an intramural fluid density collection and periaortic inflammatory changes (Fig. 1). We started empiric intravenous antibiotic therapy with meropenem 1 g intravenously (i.v.) every 8 h and vancomycin 1 g i.v. and micafungin 100 mg i.v. every 24 h. After 3 days, we assessed the effectiveness of the treatment by laboratory examination and eCT. Despite strict blood pressure control and broad-spectrum antibiotic therapy, inflammation scores became exacerbated and were represented by elevated C-reactive protein (11.61 to 28.71 mg/dl) and procalcitonin (0.34 to 0.84 ng/ml) levels and an elevated white blood cell count (9920 to 17,800/μl). In addition, the patient had a high fever (up to 39.9 °C). To prevent aneurysmal rupture and eliminate the infected foci, the patient underwent ascending aorta and aortic arch resection and subsequent total arch replacement with rifampicin-immersed artificial vessels on day five. The resected infected tissue was subjected to pathological analysis (Fig. 2), culture examination and 16S rRNA gene sequence analysis (Fig. 3) to identify the pathogen. A few days after surgical treatment, the culture and sequencing results revealed only *B. vulgatus/B. dorei* and *B. dorei* (Fig. 3, Additional files 1, and 2) as the pathogen, respectively. Based on the identification of the pathogen and on the results of susceptibility tests, the three antibiotic therapies could be discontinued and were shifted stepwise to a single metronidazole (1500 mg/day orally for 8 weeks) therapy. The patient satisfactorily improved, with no severe complications, and was transferred to another hospital for rehabilitation on postoperative day 34. There was no recurrence of infection or aneurysm within the following 12 months, as confirmed by blood tests and eCT.

## Discussion and conclusions

Anaerobic bacteria, including *Bacteroides* spp*.*, are indigenous bacteria that usually reside in the lower intestinal tract. However, they are sometimes detected as pathogens in patients with infectious diseases, especially in those with some immunosuppressive states, including in uncontrolled DM and with the use of immunosuppressive agents, such as steroids and chemotherapeutic drugs. This patient had DM with poor glycaemic control, which might explain some degree of immunosuppression and the development of the mycotic thoracic aneurysm caused by *B. dorei*.

Mycotic aneurysms per se are associated with high morbidity and mortality [9]. Moreover, anaerobic infections can be highly lethal and life threatening, and their mortality rates are estimated to be as high as 40% [10]. Therefore, it is crucial to immediately identify pathogenic bacteria and initiate appropriate antibiotic therapy targeting the identified specific pathogen. In the last decade, in addition to conventional culture tests, MALDI-TOF MS has been widely used for clinical examination. This method allows the identification of pathogens a few minutes after applying samples but has some limitations for bacteria that have similar protein compositions, as well as for uncommon bacterial species, partially due to incomplete reference databases. Because the gene sequence divergence between *B. dorei* and *B. vulgatus* is only 5% [1], two major commercially available MALDI-TOF MS systems misidentify *B. dorei* as *B. vulgatus* or cannot distinguish the two species [4, 5]. Our facility also employs a MALDI-TOF MS system for the identification of pathogens; however, this method could not discriminate between *B. dorei* and *B. vulgatus* and identified the pathogen as *B. vulgatus/B. dorei* in this case.

16S rRNA gene sequencing is a highly potent molecular biological approach for identifying specific bacteria to the species level, particularly in the case of uncommon, slow-growing or unculturable bacteria, such as minor anaerobes. Although this method does not allow the determination of antibiotic resistance, PCR and DNA sequencing are inexpensive and easily available, and thus, 16S rRNA gene sequencing has been used as a complementary examination tool for the accurate identification of bacteria and the discovery of novel bacterial species in clinical and laboratory settings [6]. In 2019, J. S. Johnson et al. reported the interspecies sequence entropy of the 16S rRNA gene, depicting that the V2, V3, V6, and V9 regions had relatively high sequence variations, and noted the validity of sub-regional sequencing for the discrimination among closely related bacteria from specific taxa [11]. In preliminary experiments, we initially amplified the full, first-half and second-half lengths of the 16S rRNA gene sequence and showed that the second-half sequence tended to be amplified more efficiently, and amplicon sequencing could satisfactorily identify specific bacteria (data not shown). In this case, by amplifying and sequencing the V5-V9 segments, we successfully identified the pathogen as *B. dorei* with 100% sequence identity with an available *B. dorei* sequence. Altogether, the data corroborated the notion that partial 16S rRNA gene sequencing, which included at least two of the aforementioned four variable regions, had sufficient capability for discriminating between specific allied bacterial species.

With the development of DNA sequence-based bacterial identification, the pathophysiology of *B. dorei* has been gradually uncovered. When we searched the PubMed database using the keyword ‘*Bacteroides dorei*’, only 50 articles were published by 1 June 2020. This bacterium is seemingly innocuous in healthy individuals, as *Bacteroidetes* and *Firmicutes* constitute over 90% of the healthy gut microbial assemblage [12]. However, it has been demonstrated that the low proportion of *B. dorei* in the gut microbiota is associated with a variety of diseases, including atherosclerotic diseases [13–16], autoimmune type 1 DM [17–22], colorectal disorders [23–26], and even Parkinson’s disease [27]. However, there are almost no reports regarding *B. dorei* as a cause of infectious diseases or even a part of the process of infection, consisting of tissue invasion, multiplication and colonisation and infliction of host tissue damage via cytotoxic mediators or direct interactions. In immunocompromised or dysbiosis states, which result in a permeable gut and impaired mucosal barriers, pathogens may invade nearby tissues or enter systemic circulation, consequently initiating infectious diseases. Although these mechanisms can be assumed, there is no sufficient evidence to understand the pathogenesis of *B. dorei* infection. Hence, this report describes the first case of an invasive infectious disease, a mycotic aneurysm, caused by *B. dorei* [9]. Further studies are needed to elucidate the process of infection.

Because these diseases are associated with dysbacteriosis or an alteration in the *B. dorei* proportion in the gut microbiota, the latter might be a target for preventative or therapeutic interventions. Some researchers have proposed using certain indigenous bacteria, including *B. dorei*, as pre−/probiotics for modulating the gut bacterial composition [8, 14, 15, 28–30]. However, as the microbiome composition is influenced by daily meals, eating habits and geography and can temporally vary even in the same individuals, the efficacy of probiotics may be condition dependent. Furthermore, as the gut microbiota forms complex systems (e.g. metabolic networks, interactions with the immune system or inter-microbial interactions), the effects of modifying the abundances of specific bacteria are not necessarily predictable [31]. Moreover, owing to its invasive potential and ability to cause infectious diseases, such as in this case report, considerable attention must be paid to the use of *B. dorei* as a probiotic. Additional studies regarding the application of probiotics or modulating strategies for the gut microbiota are needed.

The metabolic profile of *B. dorei* has also been studied and has been shown to be unique [32–35]. To date, only two bacterial species, *Eubacterium coprostanoligenes* and *B. dorei* strain D8, in a human microbial community have been identified as having cholesterol-reducing capacity [36, 37], which has been proposed to have protective roles against atherosclerosis. However, this report presented a case of an infected aortic aneurysm caused by *B. dorei*, which was detected in a surgically dissected atherosclerotic lesion. This contradictory aspect can be partially explained by the microbial metabolic features described in a report in which *Bacteroides thetaiotaomicron* was shown to selfishly or exclusively metabolise yeast mannan [38]. These results may imply that some bacteria have preferences for a specific tissue site, such as atherosclerotic lesions or microbial community sites. As *B. dorei* strains have the potential to metabolise cholesterol, they may be predisposed to colonise atherosclerotic tissues with deposited plaques of fat, cholesterol and calcium. Therefore, *B. dorei* may potentially cause mycotic aneurysms or infective endocarditis in patients with atherosclerosis. This fact also provides a warning regarding the use of *B. dorei* as a biotherapeutic, particularly in the form of live bacteria.

In conclusion, we report the first case of an invasive infectious disease caused by *B. dorei* in a patient with a mycotic thoracic aneurysm, which disagrees with the proposed protective roles of *B. dorei* in atherosclerotic diseases.

## Supplementary Information

**Additional file 1.**

**Additional file 2.**

## Data Availability

All data generated or analysed during this study are included in this published article and its additional files.

## Abbreviations

eCT: Contrast-enhanced computed tomography
i.v: Intravenously
MALDI-TOF MS: Matrix-assisted laser desorption ionisation time-of-flight mass spectrometry
PCR: Polymerase chain reaction
