# Supplementary material for: First case of an invasive Bacteroides dorei infection detected in a patient with a mycotic aortic aneurysm—raising a rebellion of major indigenous bacteria in humans: a case report and review
Source: BMC Infect Dis. 2021 Jun 30;21:625. doi: 10.1186/s12879-021-06345-8 (PMC8247135; doi:10.1186/s12879-021-06345-8)
Supplement: Supplementary file 1 — Additional file 1. [file 12879_2021_6345_MOESM1_ESM.pdf]

Additional file 1

Results of the database search using the 16S rRNA gene sequence read.

The nucleotide sequence was compared against the NCBI Bacteria & Archaea Isolate Database, with the statistical significance calculated by BLAST. The closest known bacteria are listed in the table with query cover rates, sequence identity rates and BLAST scores. BLAST: Basic Local Alignment Search Tool; NCBI: National Center for Biotechnology Information

No.	Description	Max Score	Total Score	Query Cover	E Value	Per. Ident	Accession No.
1	Bacteroides dorei strain DSM 17855 chromosome, complete genome	1271	8901	1	0	100.00%	CP046176.1
2	Bacteroides dorei strain JR01 chromosome, complete genome	1271	8901	1	0	100.00%	CP046424.1
3	Bacteroides dorei strain JR02 chromosome, complete genome	1271	8901	1	0	100.00%	CP046425.1
4	Bacteroides dorei strain JR03 chromosome, complete genome	1271	8901	1	0	100.00%	CP046426.1
5	Bacteroides dorei strain JR04 chromosome, complete genome	1271	8895	1	0	100.00%	CP046427.1
6	Bacteroides dorei strain JR05 chromosome, complete genome	1271	8895	1	0	100.00%	CP046428.1
7	Bacteroides dorei isolate MGYG-HGUT-02478 genome assembly, chromosome: 1	1271	8423	1	0	100.00%	LR699004.1
8	Bacteroides dorei strain ClaCZ110 16S ribosomal RNA gene, partial sequence	1271	1271	1	0	100.00%	MN055948.1
9	Uncultured Bacteroides sp. clone H09_plate163 16S ribosomal RNA gene, partial sequence	1271	1271	1	0	100.00%	MH515981.1
10	Uncultured Bacteroides sp. clone C05_plate163 16S ribosomal RNA gene, partial sequence	1271	1271	1	0	100.00%	MH515960.1
11	Uncultured Bacteroides sp. clone C01_plate163 16S ribosomal RNA gene, partial sequence	1271	1271	1	0	100.00%	MH515959.1
12	Uncultured Bacteroides sp. clone A01_plate163 16S ribosomal RNA gene, partial sequence	1271	1271	1	0	100.00%	MH515956.1
13	Uncultured Bacteroides sp. clone H12_plate171 16S ribosomal RNA gene, partial sequence	1271	1271	1	0	100.00%	MH515584.1
14	Uncultured Bacteroides sp. clone H11_plate171 16S ribosomal RNA gene, partial sequence	1271	1271	1	0	100.00%	MH515583.1
15	Uncultured Bacteroides sp. clone H10_plate171 16S ribosomal RNA gene, partial sequence	1271	1271	1	0	100.00%	MH515582.1
16	Uncultured Bacteroides sp. clone H09_plate171 16S ribosomal RNA gene, partial sequence	1271	1271	1	0	100.00%	MH515581.1
17	Uncultured Bacteroides sp. clone H08_plate171 16S ribosomal RNA gene, partial sequence	1271	1271	1	0	100.00%	MH515580.1
18	Uncultured Bacteroides sp. clone H07_plate171 16S ribosomal RNA gene, partial sequence	1271	1271	1	0	100.00%	MH515579.1
19	Uncultured Bacteroides sp. clone H06_plate171 16S ribosomal RNA gene, partial sequence	1271	1271	1	0	100.00%	MH515578.1
20	Uncultured Bacteroides sp. clone H05_plate171 16S ribosomal RNA gene, partial sequence	1271	1271	1	0	100.00%	MH515577.1
21	Uncultured Bacteroides sp. clone H04_plate171 16S ribosomal RNA gene, partial sequence	1271	1271	1	0	100.00%	MH515576.1
22	Uncultured Bacteroides sp. clone H03_plate171 16S ribosomal RNA gene, partial sequence	1271	1271	1	0	100.00%	MH515575.1
23	Uncultured Bacteroides sp. clone H02_plate171 16S ribosomal RNA gene, partial sequence	1271	1271	1	0	100.00%	MH515574.1
24	Uncultured Bacteroides sp. clone H01_plate171 16S ribosomal RNA gene, partial sequence	1271	1271	1	0	100.00%	MH515573.1
25	Uncultured Bacteroides sp. clone G12_plate171 16S ribosomal RNA gene, partial sequence	1271	1271	1	0	100.00%	MH515572.1
26	Uncultured Bacteroides sp. clone G11_plate171 16S ribosomal RNA gene, partial sequence	1271	1271	1	0	100.00%	MH515571.1
27	Uncultured Bacteroides sp. clone G09_plate171 16S ribosomal RNA gene, partial sequence	1271	1271	1	0	100.00%	MH515570.1
28	Uncultured Bacteroides sp. clone G08_plate171 16S ribosomal RNA gene, partial sequence	1271	1271	1	0	100.00%	MH515569.1
29	Uncultured Bacteroides sp. clone G07_plate171 16S ribosomal RNA gene, partial sequence	1271	1271	1	0	100.00%	MH515568.1
30	Uncultured Bacteroides sp. clone G06_plate171 16S ribosomal RNA gene, partial sequence	1271	1271	1	0	100.00%	MH515567.1
31	Uncultured Bacteroides sp. clone G05_plate171 16S ribosomal RNA gene, partial sequence	1271	1271	1	0	100.00%	MH515566.1
32	Uncultured Bacteroides sp. clone G04_plate171 16S ribosomal RNA gene, partial sequence	1271	1271	1	0	100.00%	MH515565.1
33	Uncultured Bacteroides sp. clone F12_plate171 16S ribosomal RNA gene, partial sequence	1271	1271	1	0	100.00%	MH515564.1
34	Uncultured Bacteroides sp. clone F11_plate171 16S ribosomal RNA gene, partial sequence	1271	1271	1	0	100.00%	MH515563.1
35	Uncultured Bacteroides sp. clone F10_plate171 16S ribosomal RNA gene, partial sequence	1271	1271	1	0	100.00%	MH515562.1
36	Uncultured Bacteroides sp. clone F09_plate171 16S ribosomal RNA gene, partial sequence	1271	1271	1	0	100.00%	MH515561.1
37	Uncultured Bacteroides sp. clone F08_plate171 16S ribosomal RNA gene, partial sequence	1271	1271	1	0	100.00%	MH515560.1
38	Uncultured Bacteroides sp. clone F07_plate171 16S ribosomal RNA gene, partial sequence	1271	1271	1	0	100.00%	MH515559.1
39	Uncultured Bacteroides sp. clone F06_plate171 16S ribosomal RNA gene, partial sequence	1271	1271	1	0	100.00%	MH515558.1
40	Uncultured Bacteroides sp. clone F05_plate171 16S ribosomal RNA gene, partial sequence	1271	1271	1	0	100.00%	MH515557.1
41	Uncultured Bacteroides sp. clone F04_plate171 16S ribosomal RNA gene, partial sequence	1271	1271	1	0	100.00%	MH515556.1
42	Uncultured Bacteroides sp. clone F03_plate171 16S ribosomal RNA gene, partial sequence	1271	1271	1	0	100.00%	MH515555.1
43	Uncultured Bacteroides sp. clone F02_plate171 16S ribosomal RNA gene, partial sequence	1271	1271	1	0	100.00%	MH515554.1
44	Uncultured Bacteroides sp. clone F01_plate171 16S ribosomal RNA gene, partial sequence	1271	1271	1	0	100.00%	MH515553.1
45	Uncultured Bacteroides sp. clone E12_plate171 16S ribosomal RNA gene, partial sequence	1271	1271	1	0	100.00%	MH515552.1
46	Uncultured Bacteroides sp. clone E11_plate171 16S ribosomal RNA gene, partial sequence	1271	1271	1	0	100.00%	MH515551.1
47	Uncultured Bacteroides sp. clone E10_plate171 16S ribosomal RNA gene, partial sequence	1271	1271	1	0	100.00%	MH515550.1
48	Uncultured Bacteroides sp. clone E09_plate171 16S ribosomal RNA gene, partial sequence	1271	1271	1	0	100.00%	MH515549.1
49	Uncultured Bacteroides sp. clone E08_plate171 16S ribosomal RNA gene, partial sequence	1271	1271	1	0	100.00%	MH515548.1
50	Uncultured Bacteroides sp. clone E07_plate171 16S ribosomal RNA gene, partial sequence	1271	1271	1	0	100.00%	MH515547.1
51	Uncultured Bacteroides sp. clone E06_plate171 16S ribosomal RNA gene, partial sequence	1271	1271	1	0	100.00%	MH515546.1
52	Uncultured Bacteroides sp. clone E05_plate171 16S ribosomal RNA gene, partial sequence	1271	1271	1	0	100.00%	MH515545.1
53	Uncultured Bacteroides sp. clone E04_plate171 16S ribosomal RNA gene, partial sequence	1271	1271	1	0	100.00%	MH515544.1
54	Uncultured Bacteroides sp. clone E03_plate171 16S ribosomal RNA gene, partial sequence	1271	1271	1	0	100.00%	MH515543.1
55	Uncultured Bacteroides sp. clone E02_plate171 16S ribosomal RNA gene, partial sequence	1271	1271	1	0	100.00%	MH515542.1
56	Uncultured Bacteroides sp. clone E01_plate171 16S ribosomal RNA gene, partial sequence	1271	1271	1	0	100.00%	MH515541.1
57	Uncultured Bacteroides sp. clone D12_plate171 16S ribosomal RNA gene, partial sequence	1271	1271	1	0	100.00%	MH515540.1
58	Uncultured Bacteroides sp. clone D11_plate171 16S ribosomal RNA gene, partial sequence	1271	1271	1	0	100.00%	MH515539.1
59	Uncultured Bacteroides sp. clone D09_plate171 16S ribosomal RNA gene, partial sequence	1271	1271	1	0	100.00%	MH515538.1
60	Uncultured Bacteroides sp. clone D08_plate171 16S ribosomal RNA gene, partial sequence	1271	1271	1	0	100.00%	MH515537.1
61	Uncultured Bacteroides sp. clone D07_plate171 16S ribosomal RNA gene, partial sequence	1271	1271	1	0	100.00%	MH515536.1
62	Uncultured Bacteroides sp. clone D05_plate171 16S ribosomal RNA gene, partial sequence	1271	1271	1	0	100.00%	MH515535.1
63	Uncultured Bacteroides sp. clone D04_plate171 16S ribosomal RNA gene, partial sequence	1271	1271	1	0	100.00%	MH515534.1
64	Uncultured Bacteroides sp. clone D03_plate171 16S ribosomal RNA gene, partial sequence	1271	1271	1	0	100.00%	MH515533.1
65	Uncultured Bacteroides sp. clone D01_plate171 16S ribosomal RNA gene, partial sequence	1271	1271	1	0	100.00%	MH515532.1
66	Uncultured Bacteroides sp. clone C12_plate171 16S ribosomal RNA gene, partial sequence	1271	1271	1	0	100.00%	MH515531.1
67	Uncultured Bacteroides sp. clone C11_plate171 16S ribosomal RNA gene, partial sequence	1271	1271	1	0	100.00%	MH515530.1
68	Uncultured Bacteroides sp. clone C10_plate171 16S ribosomal RNA gene, partial sequence	1271	1271	1	0	100.00%	MH515529.1
69	Uncultured Bacteroides sp. clone C09_plate171 16S ribosomal RNA gene, partial sequence	1271	1271	1	0	100.00%	MH515528.1
70	Uncultured Bacteroides sp. clone C08_plate171 16S ribosomal RNA gene, partial sequence	1271	1271	1	0	100.00%	MH515527.1
71	Uncultured Bacteroides sp. clone C07_plate171 16S ribosomal RNA gene, partial sequence	1271	1271	1	0	100.00%	MH515526.1
72	Uncultured Bacteroides sp. clone C06_plate171 16S ribosomal RNA gene, partial sequence	1271	1271	1	0	100.00%	MH515525.1
73	Uncultured Bacteroides sp. clone C05_plate171 16S ribosomal RNA gene, partial sequence	1271	1271	1	0	100.00%	MH515524.1
74	Uncultured Bacteroides sp. clone C03_plate171 16S ribosomal RNA gene, partial sequence	1271	1271	1	0	100.00%	MH515523.1
75	Uncultured Bacteroides sp. clone C02_plate171 16S ribosomal RNA gene, partial sequence	1271	1271	1	0	100.00%	MH515522.1
76	Uncultured Bacteroides sp. clone C01_plate171 16S ribosomal RNA gene, partial sequence	1271	1271	1	0	100.00%	MH515521.1
77	Uncultured Bacteroides sp. clone B11_plate171 16S ribosomal RNA gene, partial sequence	1271	1271	1	0	100.00%	MH515519.1
78	Uncultured Bacteroides sp. clone B10_plate171 16S ribosomal RNA gene, partial sequence	1271	1271	1	0	100.00%	MH515518.1
79	Uncultured Bacteroides sp. clone B09_plate171 16S ribosomal RNA gene, partial sequence	1271	1271	1	0	100.00%	MH515517.1
80	Uncultured Bacteroides sp. clone B08_plate171 16S ribosomal RNA gene, partial sequence	1271	1271	1	0	100.00%	MH515516.1
81	Uncultured Bacteroides sp. clone B07_plate171 16S ribosomal RNA gene, partial sequence	1271	1271	1	0	100.00%	MH515515.1
82	Uncultured Bacteroides sp. clone B06_plate171 16S ribosomal RNA gene, partial sequence	1271	1271	1	0	100.00%	MH515514.1
83	Uncultured Bacteroides sp. clone B05_plate171 16S ribosomal RNA gene, partial sequence	1271	1271	1	0	100.00%	MH515513.1
84	Uncultured Bacteroides sp. clone B04_plate171 16S ribosomal RNA gene, partial sequence	1271	1271	1	0	100.00%	MH515512.1
85	Uncultured Bacteroides sp. clone B03_plate171 16S ribosomal RNA gene, partial sequence	1271	1271	1	0	100.00%	MH515511.1
86	Uncultured Bacteroides sp. clone B02_plate171 16S ribosomal RNA gene, partial sequence	1271	1271	1	0	100.00%	MH515510.1
87	Uncultured Bacteroides sp. clone B01_plate171 16S ribosomal RNA gene, partial sequence	1271	1271	1	0	100.00%	MH515509.1
88	Uncultured Bacteroides sp. clone A12_plate171 16S ribosomal RNA gene, partial sequence	1271	1271	1	0	100.00%	MH515508.1
89	Uncultured Bacteroides sp. clone A11_plate171 16S ribosomal RNA gene, partial sequence	1271	1271	1	0	100.00%	MH515507.1
90	Uncultured Bacteroides sp. clone A09_plate171 16S ribosomal RNA gene, partial sequence	1271	1271	1	0	100.00%	MH515506.1
91	Uncultured Bacteroides sp. clone A08_plate171 16S ribosomal RNA gene, partial sequence	1271	1271	1	0	100.00%	MH515505.1
92	Uncultured Bacteroides sp. clone A07_plate171 16S ribosomal RNA gene, partial sequence	1271	1271	1	0	100.00%	MH515504.1
93	Uncultured Bacteroides sp. clone A06_plate171 16S ribosomal RNA gene, partial sequence	1271	1271	1	0	100.00%	MH515503.1
94	Uncultured Bacteroides sp. clone A05_plate171 16S ribosomal RNA gene, partial sequence	1271	1271	1	0	100.00%	MH515502.1
95	Uncultured Bacteroides sp. clone A03_plate171 16S ribosomal RNA gene, partial sequence	1271	1271	1	0	100.00%	MH515501.1
96	Uncultured Bacteroides sp. clone A02_plate171 16S ribosomal RNA gene, partial sequence	1271	1271	1	0	100.00%	MH515500.1
97	Uncultured Bacteroides sp. clone A01_plate171 16S ribosomal RNA gene, partial sequence	1271	1271	1	0	100.00%	MH515499.1
98	Uncultured Bacteroides sp. clone H12_plate165 16S ribosomal RNA gene, partial sequence	1271	1271	1	0	100.00%	MH515498.1
99	Uncultured Bacteroides sp. clone H11_plate165 16S ribosomal RNA gene, partial sequence	1271	1271	1	0	100.00%	MH515497.1
100	Uncultured Bacteroides sp. clone H10_plate165 16S ribosomal RNA gene, partial sequence	1271	1271	1	0	100.00%	MH515496.1
