# Supplementary material for: First case of an invasive Bacteroides dorei infection detected in a patient with a mycotic aortic aneurysm—raising a rebellion of major indigenous bacteria in humans: a case report and review
Source: BMC Infect Dis. 2021 Jun 30;21:625. doi: 10.1186/s12879-021-06345-8 (PMC8247135; doi:10.1186/s12879-021-06345-8)
Supplement: Supplementary file 2 — Additional file 2. [file 12879_2021_6345_MOESM2_ESM.pdf]

Additional file 2

Alignment of the 16S rRNA gene sequence from the type strain of *Bacteroides dorei* (DSM 17855) and that obtained by Sanger sequencing from the patient's specimen. Total genomic DNA was purified from the patient's infected aortic wall and subjected to PCR amplification using the 500F (5′-GGATTAGATACCCTGGTA-3′) and 1500R (5′-TACCTTGTTACGACTT-3′) universal primers. Sanger sequencing of the amplicon was performed, and the sequence obtained for the single-peak electropherogram read was compared with that of the type strain of *B. dorei* (DSM 17855). The 500F and 1500R primers are shown as red arrows, and the aligned sequence is provided below the *B. dorei* 16S rRNA gene sequence. The full-length sequence from the V5 to V9 segments completely coincides with the corresponding sequence of *B. dorei*.

```
1 ATTTTTACAA TGAAGAGTTT GATCCTGGCT CAGGATGAAC GCTAGCTACA GGCTTAACAC 60
61 ATGCAAGTCG AGGGGCAGCA TGGTCTTAGC TTGCTAAGGC TGATGGCGAC CGGCGCACGG 120
121 GTGAGTAACA CGTATCCAAC CTGCCGTCTA CTCTTGGCCA GCCTTCTGAA AGGAAGATTA 180
181 ATCCAGGATG GGATCATGAG TTCACATGTC CGCATGATTA AAGGTATTTT CCGGTAGACG 240
241 ATGGGGATGC GTTCCATTAG ATAGTAGGCG GGGTAACGGC CCACCTAGTC AACGATGGAT 300
301 AGGGGTTCTG AGAGGAAGGT CCCCCACATT GGAACTGAGA CACGGTCCAA ACTCCTACGG 360
361 GAGGCAGCAG TGAGGAATAT TGGTCAATGG GCGATGGCCT GAACCAGCCA AGTAGCGTGA 420
421 AGGATGACTG CCCTATGGGT TGTAAACTTC TTTTATAAAG GAATAAAGTC GGGTATGCAT 480
481 ACCCGTTTGC ATGTACTTTA TGAATAAGGA TCGGCTAACT CCGTGCCAGC AGCCGCGGTA 540
541 ATACGGAGGA TCCGAGCGTT ATCCGGATTT ATTGGGTTTA AAGGGAGCGT AGATGGATGT 600
601 TTAAGTCAGT TGTGAAAGTT TGCGGCTCAA CCGTAAAATT GCAGTTGATA CTGGATGTCT 660
661 TGAGTGCAGT TGAGGCAGGC GGAATTCGTG GTGTAGCGGT GAAATGCTTA GATATCACGA 720
721 AGAACTCCGA TTGCGAAGGC AGCCTGCTAA GCTGCAACTG ACATTGAGGC TCGAAAGTGT 780

                         800F primer
781 GGGTATCAAA CAGGATTAGA TACCCTGGTA GTCCACACGG TAAACGATGA ATACTCGCTG 840
                             CTGGTA GTCCACACGG TAAACGATGA ATACTCGCTG

841 TTTGCGATAT ACGGCAAGCG GCCAAGCGAA AGCGTTAAGT ATTCCACCTG GGGAGTACGC 900
    TTTGCGATAT ACGGCAAGCG GCCAAGCGAA AGCGTTAAGT ATTCCACCTG GGGAGTACGC

901 CGGCAACGGT GAAACTCAAA GGAATTGACG GGGGCCCGCA CAAGCGGAGG AACATGTGGT 960
    CGGCAACGGT GAAACTCAAA GGAATTGACG GGGGCCCGCA CAAGCGGAGG AACATGTGGT

961 TTAATTCGAT GATACGCGAG GAACCTTACC CGGGCTTAAA TTGCACTCGA ATGATCCGGA 1020
    TTAATTCGAT GATACGCGAG GAACCTTACC CGGGCTTAAA TTGCACTCGA ATGATCCGGA

1021 AACGGTTCAG CTAGCAATAG CGAGTGTGAA GGTGCTGCAT GGTTGTCGTC AGCTCGTGCC 1080
     AACGGTTCAG CTAGCAATAG CGAGTGTGAA GGTGCTGCAT GGTTGTCGTC AGCTCGTGCC

1081 GTGAGGTGTC GGCTTAAGTG CCATAACGAG CGCAACCCTT GTTGTCAGTT ACTAACAGGT 1140
     GTGAGGTGTC GGCTTAAGTG CCATAACGAG CGCAACCCTT GTTGTCAGTT ACTAACAGGT

1141 GATGCTGAGG ACTCTGACAA GACTGCCATC GTAAGATGTG AGGAAGGTGG GGATGACGTC 1200
     GATGCTGAGG ACTCTGACAA GACTGCCATC GTAAGATGTG AGGAAGGTGG GGATGACGTC

1201 AAATCAGCAC GGCCCTTACG TCCGGGGCTA CACACGTGTT ACAATGGGGG GTACAGAGGG 1260
     AAATCAGCAC GGCCCTTACG TCCGGGGCTA CACACGTGTT ACAATGGGGG GTACAGAGGG

1261 CCGCTACCAC GCGAGTGGAT GCCAATCCCT AAAACCCCTC TCAGTTCGGA CTGGAGTCTG 1320
     CCGCTACCAC GCGAGTGGAT GCCAATCCCT AAAACCCCTC TCAGTTCGGA CTGGAGTCTG

1321 CAACCCGACT CCACGAAGCT GGATTCGCTA GTAATCGCGC ATCAGCCACG GCGCGGTGAA 1380
     CAACCCGACT CCACGAAGCT GGATTCGCTA GTAATCGCGC ATCAGCCACG GCGCGGTGAA

1381 TACGTTCCCG GGCCTTGTAC ACACCGCCCG TCAAGCCATG GGAGCCGGGG GTACCTGAAG 1440
     TACGTTCCCG GGCCTTGTAC ACACCGCCCG TCAAGCCATG GGAGCCGGGG GTACCTGAAG

                                                            1500R primer
1441 TGCGTAACCG CGAGGATCGC CCTAGGGTAA AACTGGTGAC TGGGGCTAAG TCGTAACAAG 1500
     TGCGTAACCG CGAGGATCGC CCTAGGGTAA AACTGGTGAC TGGGGCTAAG TC

1501 GTAGCCGTAC CGGAAGGTGC GGCTGGAACA CCTCCTTTCT 1540
```
